# Novel post-transcriptional and post-translational regulation of pro-apoptotic protein BOK and anti-apoptotic protein Mcl-1 determine the fate of breast cancer cells to survive or die

**DOI:** 10.18632/oncotarget.20841

**Published:** 2017-09-12

**Authors:** Benjamin Onyeagucha, Panneerdoss Subbarayalu, Nourhan Abdelfattah, Subapriya Rajamanickam, Santosh Timilsina, Rosa Guzman, Carla Zeballos, Vijay Eedunuri, Sanjay Bansal, Tabrez Mohammad, Yidong Chen, Ratna K. Vadlamudi, Manjeet K. Rao

**Affiliations:** ^1^ Greehey Children’s Cancer Research Institute, The University of Texas Health Science Center at San Antonio, Texas, 78229 USA; ^2^ Department of Cell Systems and Anatomy, The University of Texas Health Science Center at San Antonio, Texas, 78229 USA; ^3^ Department of Epidemiology and Statistics, The University of Texas Health Science Center at San Antonio, Texas, 78229 USA; ^4^ Department of Obstetrics and Gynecology, The University of Texas Health Science Center at San Antonio, Texas, 78229 USA

**Keywords:** breast cancer, apoptosis, BOK, Mcl-1, GSK3α/β

## Abstract

Deregulation of apoptosis is central to cancer progression and a major obstacle to effective treatment. The Bcl-2 gene family members play important roles in the regulation of apoptosis and are frequently altered in cancers. One such member is pro-apoptotic protein Bcl-2-related Ovarian Killer (BOK). Despite its critical role in apoptosis, the regulation of BOK expression is poorly understood in cancers. Here, we discovered that miR-296-5p regulates BOK expression by binding to its 3’-UTR in breast cancers. Interestingly, miR-296-5p also regulates the expression of anti-apoptotic protein myeloid cell leukemia 1 (Mcl-1), which is highly expressed in breast cancers. Our results reveal that Mcl-1 and BOK constitute a regulatory feedback loop as ectopic BOK expression induces Mcl-1, whereas silencing of Mcl-1 results in reduced BOK levels in breast cancer cells. In addition, we show that silencing of Mcl-1 but not BOK reduced the long-term growth of breast cancer cells. Silencing of both Mcl-1 and BOK rescued the effect of Mcl-1 silencing on breast cancer cell growth, suggesting that BOK is important for attenuating cell growth in the absence of Mcl-1. Depletion of BOK suppressed caspase-3 activation in the presence of paclitaxel and in turn protected cells from paclitaxel-induced apoptosis. Furthermore, we demonstrate that glycogen synthase kinase (GSK3) α/β interacts with BOK and regulates its level post-translationally in breast cancer cells. Taken together, our results suggest that fine tuning of the levels of pro-apoptotic protein BOK and anti-apoptotic protein Mcl-1 may decide the fate of cancer cells to either undergo apoptosis or proliferation.

## INTRODUCTION

Apoptosis is an evolutionary conserved process that is critical for the maintenance of tissue homeostasis in multicellular organisms [1]. Apoptosis also plays an important role during embryonic development [2, 3]. In addition, alteration of apoptotic pathways is associated with several pathological conditions including cancers [4–6]. For example, evasion of apoptosis is central to cancer growth and progression and defects in apoptotic pathways result in resistance to chemotherapy drug response [7, 8]. There are two major apoptotic pathways: extrinsic pathway, which involves transmembrane death receptor-mediated interactions and intrinsic pathway, which involves mitochondrial-dependent events. The Bcl-2 gene family members are key regulators of intrinsic apoptotic pathway that include pro- and anti-apoptotic proteins [9, 10]. Examples of anti-apoptotic proteins include Bcl-2, Bcl-xL, and Mcl-1, that are important for maintaining the mitochondrial integrity, while BAX, BAK, BAD and BOK are pro-apoptotic proteins that facilitate the disruption and release of cytochrome c, an apoptogenic factor from the intermembrane space of the mitochondria, crucial for the activation of caspase-3 and caspase-7 that execute the final steps of programmed cell death [11]. Bcl-2 gene family members are known to be important contributors to tumorigenesis and therefore, are considered as promising therapeutic targets [12]. For example, Bcl-2 and Mcl-1 are highly expressed in cancers, promote cancer cell survival and are associated with poor therapeutic outcomes [13, 14]. In addition, increased levels of Bcl-2 or Mcl-1 promote accumulation of apoptotic resistant neoplastic cells and also help cancer cells evade immune-surveillance [15]. Similarly, loss of pro-apoptotic proteins such as BAX or BAK is frequently observed in cancers [16]. Despite their proven role in tumorigenesis and years of investigation, we are far from completely understanding the mechanism(s) by which anti-apoptotic and pro-apoptotic proteins are regulated in cancers. It is especially important given that the dynamic equilibrium of pro-apoptotic and anti-apoptotic proteins is crucial for maintaining cellular homeostasis and disruption of this balance is one of the ways cancer cells can evade apoptosis.

In this report, we address the regulation of pro-apoptotic protein BOK in breast cancers. BOK is localized in the mitochondria, endoplasmic reticulum (ER), Golgi, and nucleus [17]. The pro-apoptotic function of BOK has been shown to be largely dependent on the presence of BAX or BAK. In addition, BOK is reported to interact with anti-apoptotic protein Mcl-1 and BOK-induced apoptosis is shown to be suppressed by Mcl-1 [17, 18]. In addition, recent evidence shows that BOK is frequently deleted in cancers [19]. Our results reveal that BOK expression in breast cancer is regulated at the post-transcriptional as well as post-translational levels. We show that miR-296-5p regulates BOK expression by binding to its 3’ untranslated region (UTR). Interestingly, we show that miR-296-5p also regulates Mcl-1 expression by binding to its 3’-UTR. Furthermore, we report that BOK and Mcl-1 may constitute a feedback loop to regulate each other’s stability and function. More notably, knockdown of BOK attenuates paclitaxel-induced caspase-3 activation and subsequently apoptosis. In addition to miR-296-5p, we demonstrate that glycogen synthase kinases 3 α/β (GSK3α/β) regulate BOK expression by physically interacting with BOK in breast cancer cells. These findings suggest that the levels of pro-apoptotic and anti-apoptotic signals are tightly regulated and any alteration in the relative ratio and function of pro-and anti-apoptotic proteins can predispose a normal developmental event into malignant transformation.

## RESULTS

### Decreased BOK level in breast cancers

**Figure 1:**
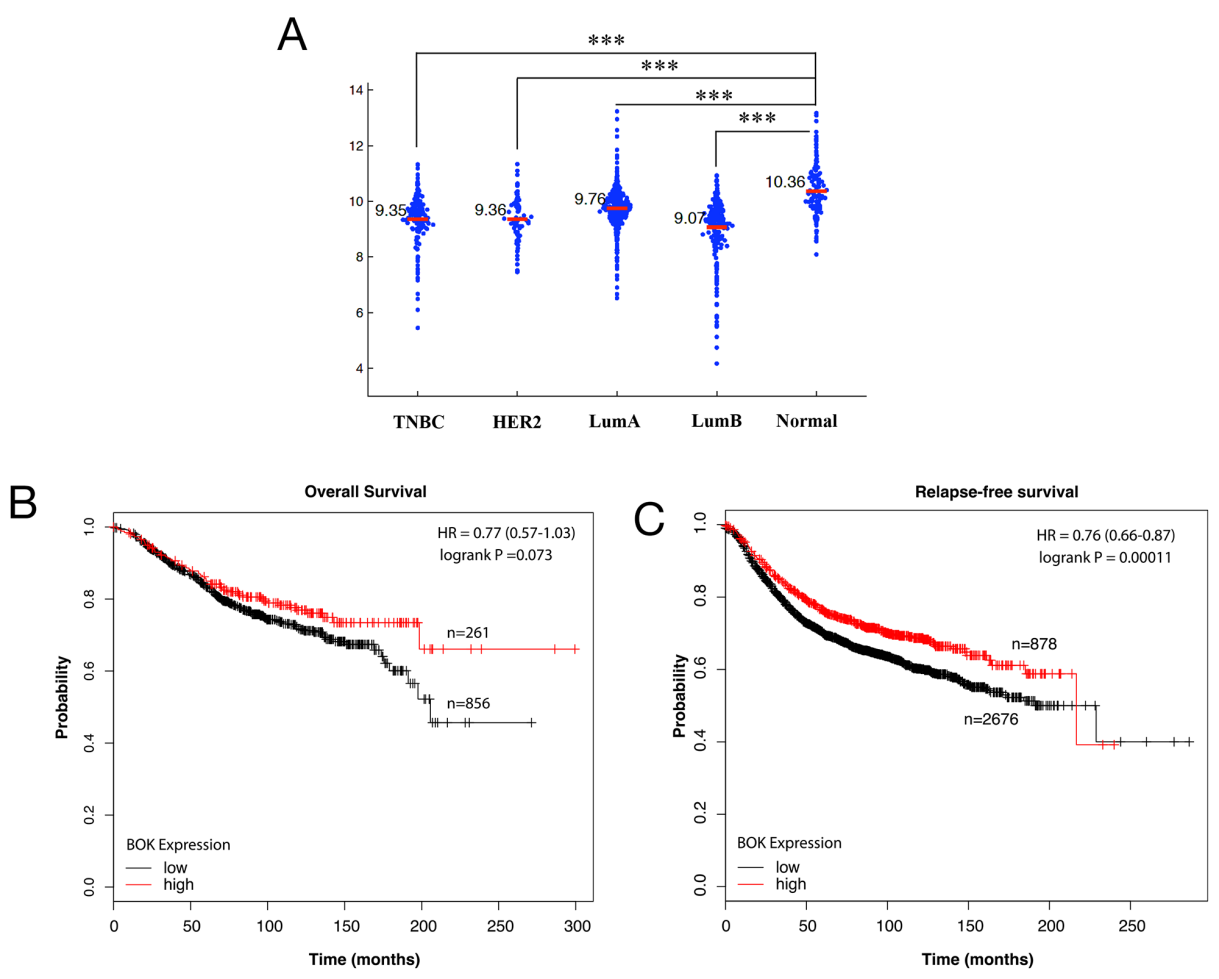
BOK expression is decreased in breast cancers **(A)** Meta-analysis of BOK expression using TCGA data set for breast cancers and normal tissues. **(B, C)** Kaplan-Meier analyses of overall survival and relapse-free survival of breast cancer patients using KM plotter database (www.kmplot.com) [44]. The *p-values* were calculated with logrank (Mantel-Cox) test. Patients were stratified into ‘low’ and ‘high’ BOK expression based on upper quartile as cutoff. The results shown here are in whole based upon data generated by the TCGA Research Network: http://cancergenome.nih.gov/. *** *p*<0.0001.

To begin to address the importance of BOK in cancers, we first investigated BOK expression levels in breast cancers. Meta-analysis of a Cancer Genome Atlas (TCGA) data set for breast cancers (https://gdc.cancer.gov/) [20] showed significantly lower levels of BOK in tumor tissue specimens compared to normal controls (Figure 1A). Next, we addressed the clinical significance of lower BOK expression in breast cancers. Kaplan-Meir analysis revealed that higher BOK expression positively correlated with overall survival as well as relapse free survival of breast cancer patients (Figures 1B–1C).

### miR-296-5p regulates both pro-apoptotic protein BOK and anti-apoptotic protein Mcl-1

**Figure 2:**
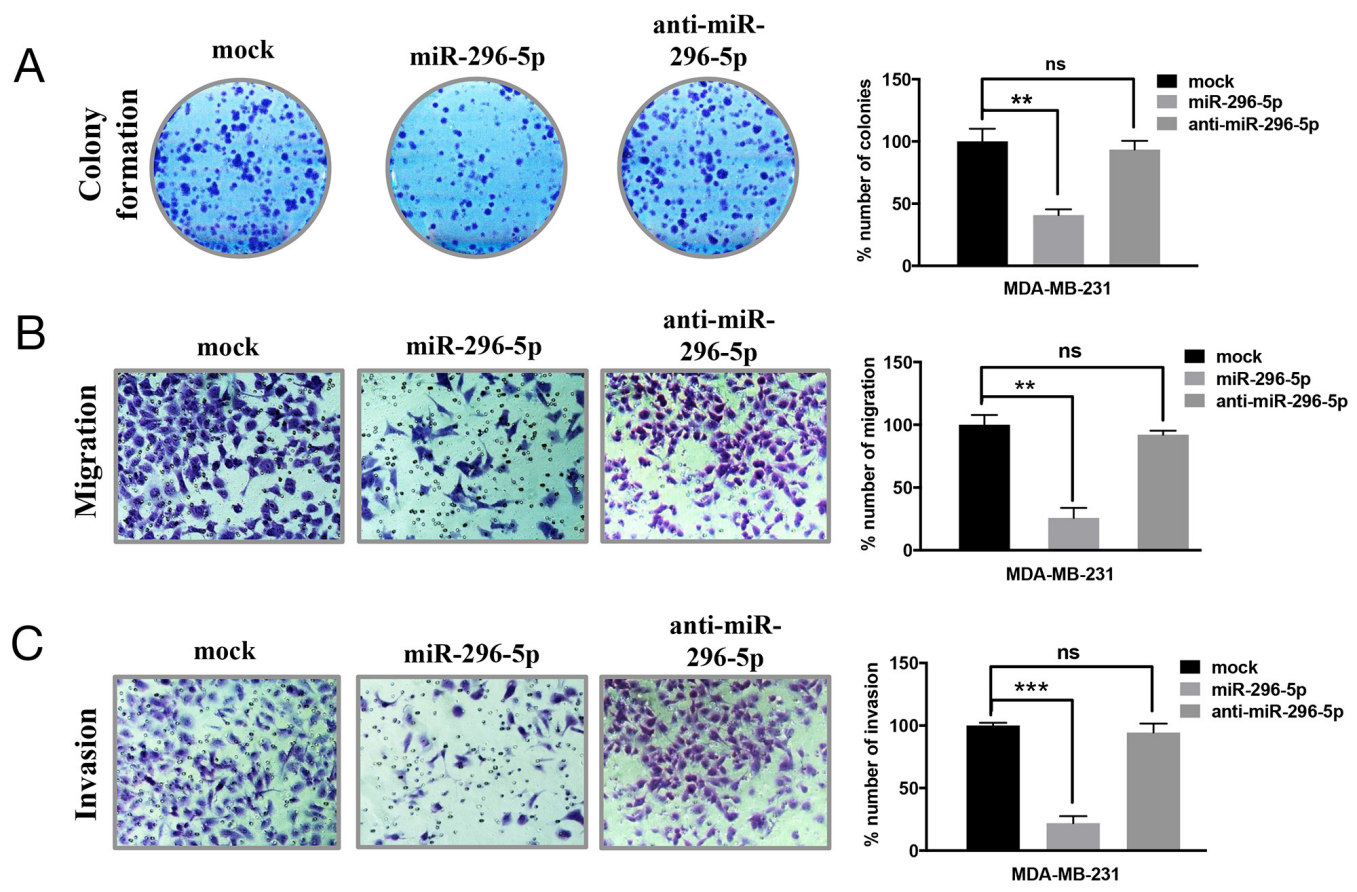
miR-296-5p inhibits long-term viability, migration and invasion of breast cancer cells **(A)** Clonogenic assay on mock, miR-296-5p or miR-296-5p inhibitor (anti-miR-296-5p)-transfected MDA-MB-231 cells. Bar graph shows number of colonies counted microscopically in ten different fields. **(B, C)** Photomicrographs showing migrated (B) and invaded (C) MDA-MB-231 cells transfected with mock, miR-296-5p or miR-296-5p inhibitor (anti-miR-296-5p). Bar graphs show number of migrated and invaded cells. The data shown are mean ± SD for at least three independent experiments. ***, *p*<0.001; **, *p*<0.01 versus control group, ANOVA.

**Figure 3:**
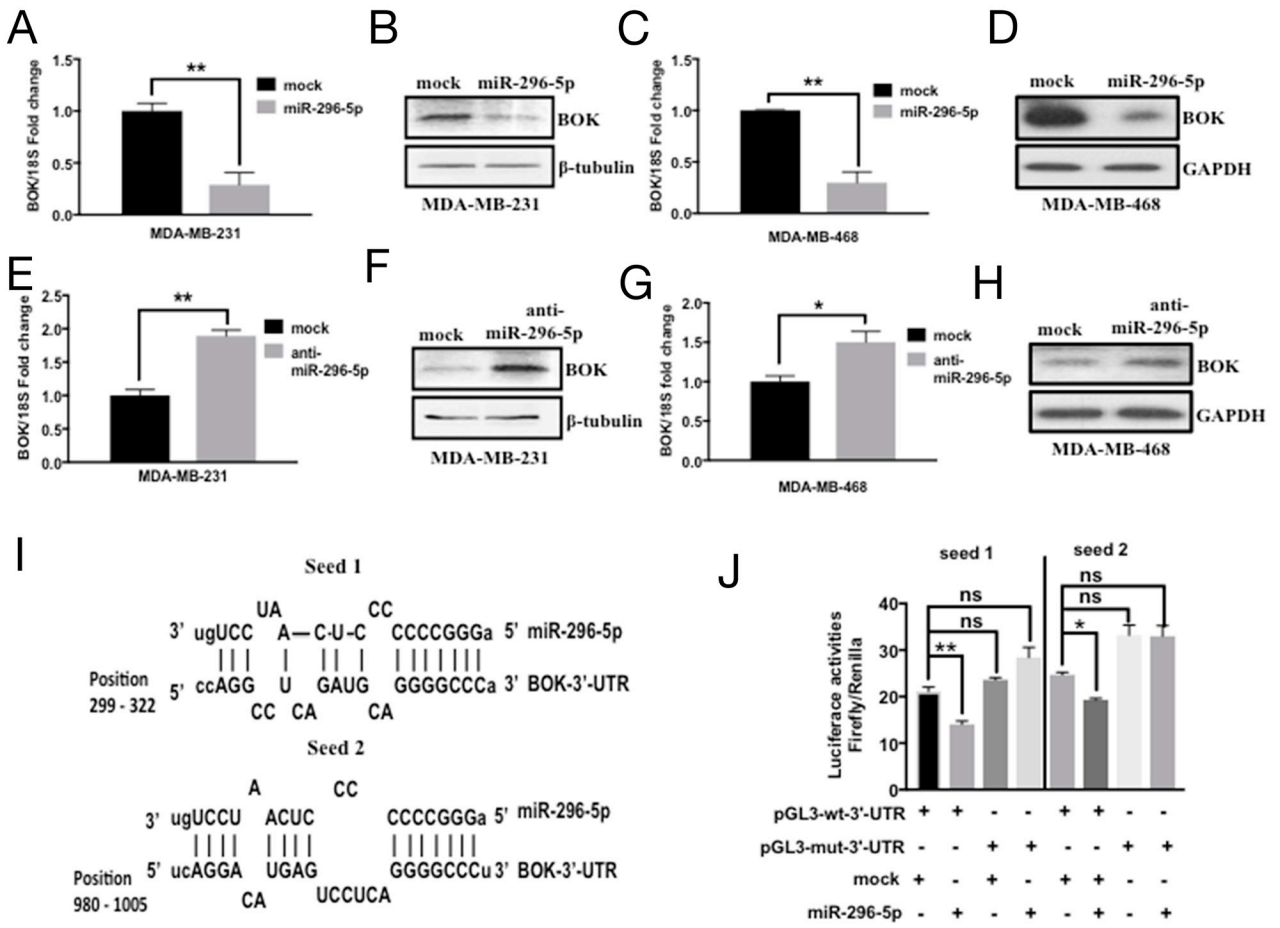
miR-296-5p regulates BOK expression in breast cancer **(A, B)** qRT-PCR (A) and Western blot (B) analysis of MDA-MB-231 cells transfected with mock or miR-296-5p using BOK-specific primers and antibody against BOK. **(C, D)** qRT-PCR (C) and Western blot (D) analysis on MDA-MB-468 cells transfected with mock or miR-296-5p using BOK-specific primers and antibody against BOK. **(E, F)** qRT-PCR (E) and Western blot (F) analysis on MDA-MB-231 cells transfected with mock or anti-miR-296-5p (miR-296-5p inhibitor) using BOK-specific primers and antibody against BOK. **(G, H)** qRT-PCR (G) and Western blot (H) analysis on MDA-MB-468 cells transfected with mock or miR-296-5p inhibitor (anti-miR-296-5p) using BOK-specific primers and antibody against BOK. Gel photographs in B, D, F and H are representative of three independent experiments. GAPDH and β-tubulin were used as loading controls. **(I)** Schematic of the putative miR-296-5p binding sequences in BOK 3’-UTR region. **(J)** Bar graph showing luciferase activity in MDA-MB-231 cells co-transfected with renilla luciferase construct (pRL-null vector) and firefly luciferase constructs containing either pGL3-wt-BOK or pGL3-BOK mutants (seed sequence 1 and seed sequence 2 mutants (seed 1, seed 2) in the presence and absence of miR-295-5p. Firefly luciferase activity for each sample was normalized with renilla luciferase activity. Data represent the mean ± SD of three independent experiments. *, *p*<0.05; **, *p*<0.01

Since miRNAs have been shown to regulate the expression of several genes involved in apoptosis, we predicted that miRNAs might play important role in regulating BOK expression in breast cancer. Using bioinformatics approach, we identified 39 miRNAs that are predicted to target BOK. Of those, miR-296-5p was predicted by at least four algorithms including Targetscan, miRDB, miRanda and mirTarget2 to bind to multiple sites in the 3’-UTR of BOK. Before we addressed miR-296-5p-dependent regulation of BOK, we determined the role of miR-296-5p in breast cancer. Consistent with the previous reports [21, 22], our results revealed that miR-296-5p acts as a tumor suppressor, as miR-296-5p inhibited long-term viability, migration as well as invasion of breast cancer cells (Figure 2). Next, we tested whether BOK is indeed a bonafide target of miR-296-5p by assessing BOK levels in MDA-MB-231 and MDA-MB-468 breast cancer cells, ectopically expressing miR-296-5p or anti-miR-296-5p (miR-296-5p inhibitor). Overexpression of miR-296-5p resulted in significantly decreased BOK mRNA and protein levels in breast cancer cells compared to mock or untransfected controls (Figures 3A–3D and Supplementary Figure 1). Conversely, inhibition of miR-296-5p using anti-miR-296-5p led to increased level of BOK (Figures 3E–3H). Next, we tested whether miR-296-5p regulates BOK expression by binding to its 3’-UTR. To examine this, we cloned two miR-296-5p predicted binding seed regions (seed 1: 299 to 322 nucleotides and seed 2: 980 to 1005 nucleotides) in the BOK 3’-UTR and their respective mutant variants downstream of luciferase gene in pGL3-promoter vector and measured the luciferase activity (Figure 3I). Co-transfection of BOK 3’-UTR constructs and miR-296-5p resulted in decreased luciferase activity when compared to mock transfected breast cancer cells (Figure 3J). In contrast, expression of the mutant BOK 3’-UTR constructs were unaffected by ectopic miR-296-5p expression (Figure 3J), further supporting the notion that BOK is a direct target of miR-296-5p.

**Figure 4:**
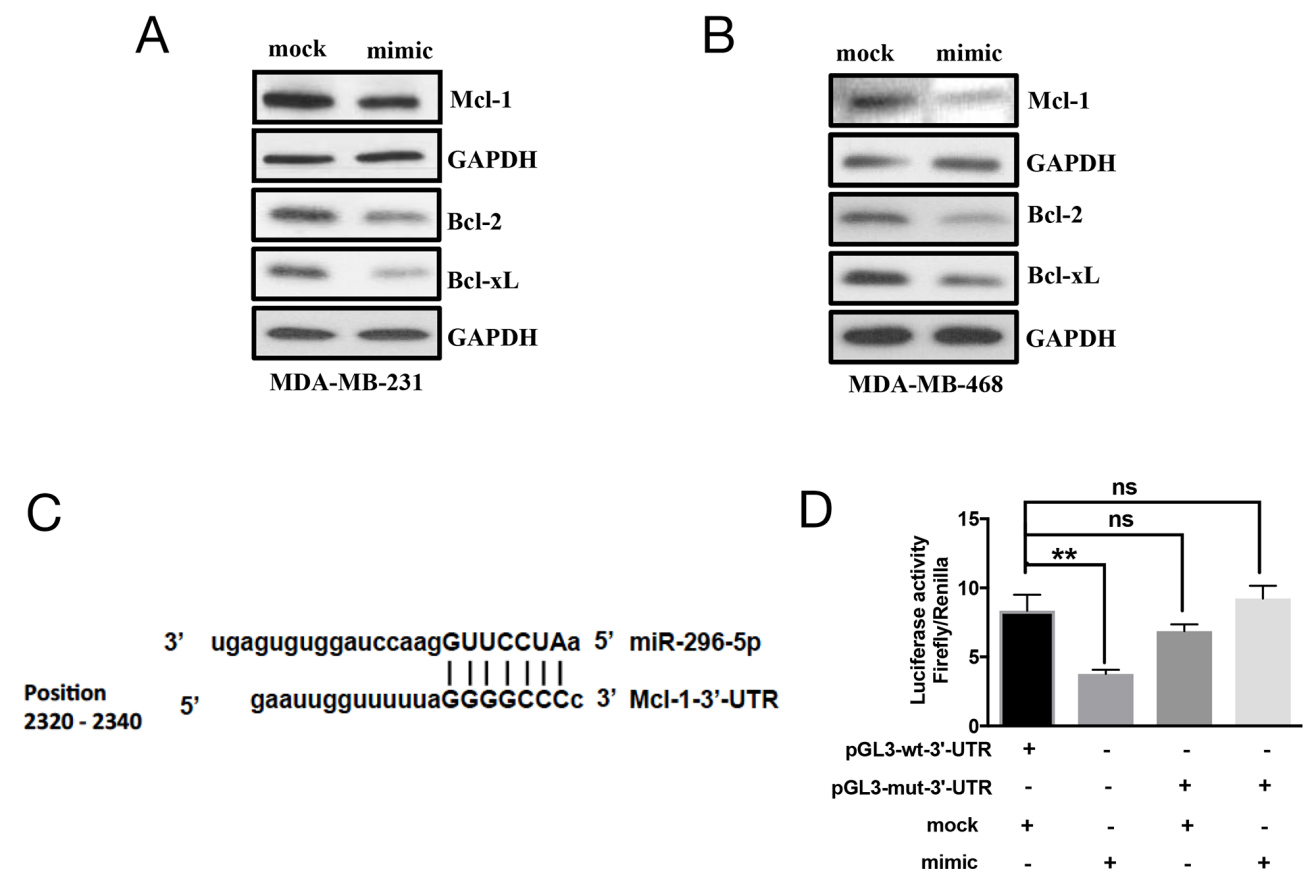
Mcl-1 is a direct target of miR-296-5p **(A, B)** Western blot analysis on MDA-MB-231 (A) and MDA-MB-468 (B) cells transfected with mock or miR-296-5p using antibodies against indicated proteins. Gel photograph is representative of three independent experiments. **(C)** Schematic of putative miR-296-5p binding site in Mcl-1 3’-UTR. **(D)** Bar graph showing luciferase activity in MDA-MB-231 co-transfected with renilla luciferase construct (pRL-null vector) and firefly luciferase constructs containing either pGL3-wt-Mcl-1 or pGL3-Mcl-1 mutant sequences (pGL-mut-3’-UTR) in the presence and absence of miR-295-5p. Firefly luciferase activity for each sample was normalized with renilla luciferase activity. Data represent the mean ± SD of three independent experiments. *, *p*<0.05; **, *p*<0.01.

As BOK pro-apoptotic function is shown to be dependent on BAX or BAK [23], we wondered whether miR-296-5p, in addition to BOK, regulates other Bcl-2 family proteins. Our bioinformatics analysis using target prediction algorithms revealed putative miR-296-5p binding sites in BID, BIM, BAK, PUMA, Mcl-1, Bcl-2, Bcl-xL 3’-UTRs (data not shown). The overexpression of miR-296-5p resulted in significantly decreased levels of Mcl-1, Bcl-2, and Bcl-xL (Figures 4A–4B and Supplementary Figure 2), however the levels of pro-apoptotic proteins BID, BIM, BAK, BAX, or PUMA did not change (Supplementary Figure 3). These findings are interesting given that Mcl-1, Bcl-2, and Bcl-xL are anti-apoptotic proteins. To determine whether anti-apoptotic proteins are directly targeted by miR-296-5p, we focused on Mcl-1 as it has been reported to interact with BOK. Moreover, our bioinformatics analysis revealed a perfect miR-296-5p seed sequence match within Mcl-1 3’-UTR sequences (Figure 4C). Co-transfection of Mcl-1-3’-UTR luciferase construct and miR-296-5p led to significantly reduced levels of luciferase activities compared to mock transfected breast cancer cells (Figure 4D). Consistent with that, mutant Mcl-1 3’-UTR (with seed sequence mutated) luciferase construct did not show any significant effect on luciferase levels in the presence of miR-295-5p (Figure 4D). We chose to use MDA-MB-231 and MDA-MB-468 cells for determining miR-296-5p function as well as regulation of BOK and Mcl-1 as these cell lines have lower levels of miR-296-5p and higher levels of BOK and Mcl-1 compared to other breast cancer cell lines (Supplementary Figures 4A–4B). Taken together, our data suggest that miR-296-5p regulates the expression of both pro-apoptotic and anti-apoptotic proteins in breast cancers.

### Functional interaction between BOK and Mcl-1

**Figure 5:**
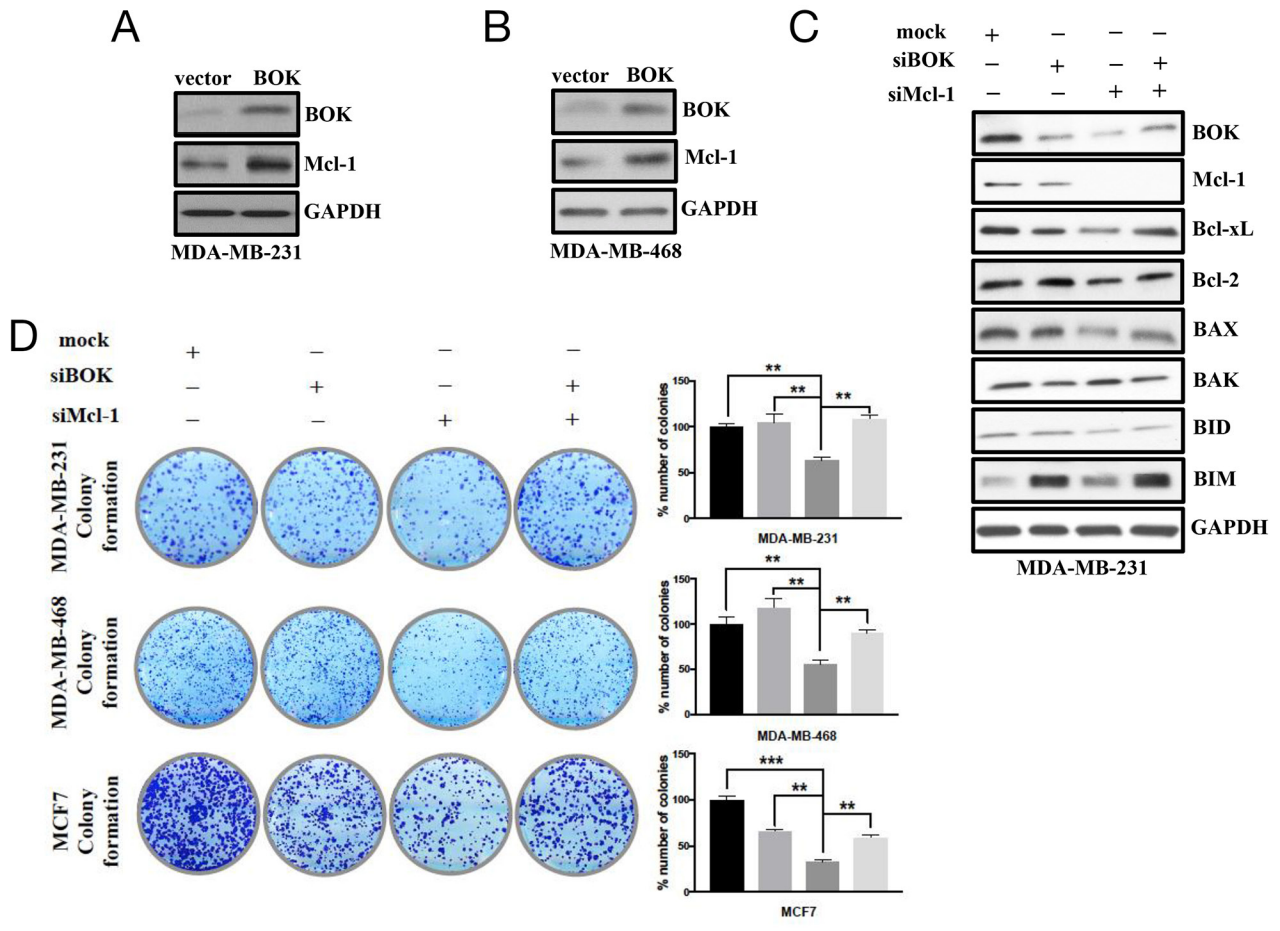
Cross-talk between BOK and Mcl-1 regulates cancer cell survival/apoptosis **(A, B)** Western blot analysis on MDA-MB-231 (A) and MDA-MB-468 (B) cells transfected with BOK expression vector using antibodies against BOK or Mcl-1. GAPDH was used as a loading control. Gel picture is representative of three independent experiments. **(C)** Western blot analysis of anti-apoptotic and pro-apoptotic proteins in mock, BOK-siRNA and Mcl-1-siRNA transfected MDA-MB-231 cells. GAPDH was used as a loading control. Gel photograph is representative of three independent experiments. **(D)** Clonogenic assay on mock, BOK-siRNA, Mcl-1-siRNA or BOK-siRNA + Mcl-1-siRNA transfected MDA-MB-231 cells. Bar graph shows number of colonies counted microscopically in ten different fields. ***, *p*<0.001; **, *p*<0.01; *, *p*<0.05.

BOK was initially identified as a Mcl-1 interacting protein in a Yeast Two-Hybrid system [24]. Here, we evaluated whether BOK-Mcl-1 interaction affected their levels and respective functions in breast cancers. Indeed, ectopic expression of BOK induced Mcl-1 expression (Figures 5A, 5B), while silencing of Mcl-1 resulted in reduced expression of BOK in breast cancer cells (Figure 5C). Interestingly, Our results showing BOK and Mcl-1 functional interaction and regulation of both BOK and Mcl-1 by miR-296-5p suggest that levels of pro-apoptotic and anti-apoptotic proteins must be in a dynamic equilibrium. To substantiate this notion, we tested the expression of other pro- and anti-apoptotic members of Bcl-2 family in BOK/Mcl-1 silenced breast cancer cells. Knockdown of either BOK or Mcl-1 resulted in significantly reduced levels of anti-apoptotic Bcl-xL, Bcl-2 proteins and also pro-apoptotic proteins BAK and BAX (Figure 5C, Supplementary Figure 5). Surprisingly, knockdown of both BOK and Mcl-1 increased the levels of Bcl-xL, Bcl-2, BIM, and BAX when compared to either BOK or Mcl-1 knockdown alone. These findings indicated that the pro- and anti-apoptotic proteins regulate each other’s expression in cancer cells. To understand the functional relevance of the cross-talk between pro- and anti-apoptotic proteins, we performed long-term viability assay in breast cancer cells depleted for both BOK/Mcl-1. Our result showed that knockdown of BOK has no effect on long-term viability, while silencing of Mcl-1 led to significantly reduced number of colonies in MDA-MB-231, MDA-MB-468, and MCF7 breast cancer cells (Figure 5D). However, the number of MCF7 colonies was significantly reduced in both BOK and Mcl-1-silenced cells (Figure 5D). The reduced long-term survival of BOK-silenced MCF7 cells could be due to higher levels of miR-296-5p in MCF7 cells compared to MDA-MB-231 and MDA-MB-468 cells. In addition, the presence of functional p53 along with positive ER status of MCF7 cells [25] could affect its long-term survival in BOK depleted condition. In accordance with this, ERα is known to activate p53 by blocking MDM2 inhibition of p53 [26]. Therefore, it is possible that presence of ERα may amplify p53 stability and increased activation of p53 may sensitize cells to apoptotic signaling in BOK-silenced MCF7 cells. Furthermore, differences in expression of Bcl-2 family proteins and lack of functional caspase-3 in MCF7 cells [27] may also contribute to reduce long-term viability of BOK-silenced MCF7 cells compared to MDA-MB-231 or MDA-MB-468 cells. Nevertheless, depletion of both BOK and Mcl-1 rescued the effect of Mcl-1 silencing on long-term viability of MDA-MB-231, MDA-MB-468 and MCF7 breast cancer cells (Figure 5D).

**Figure 6:**
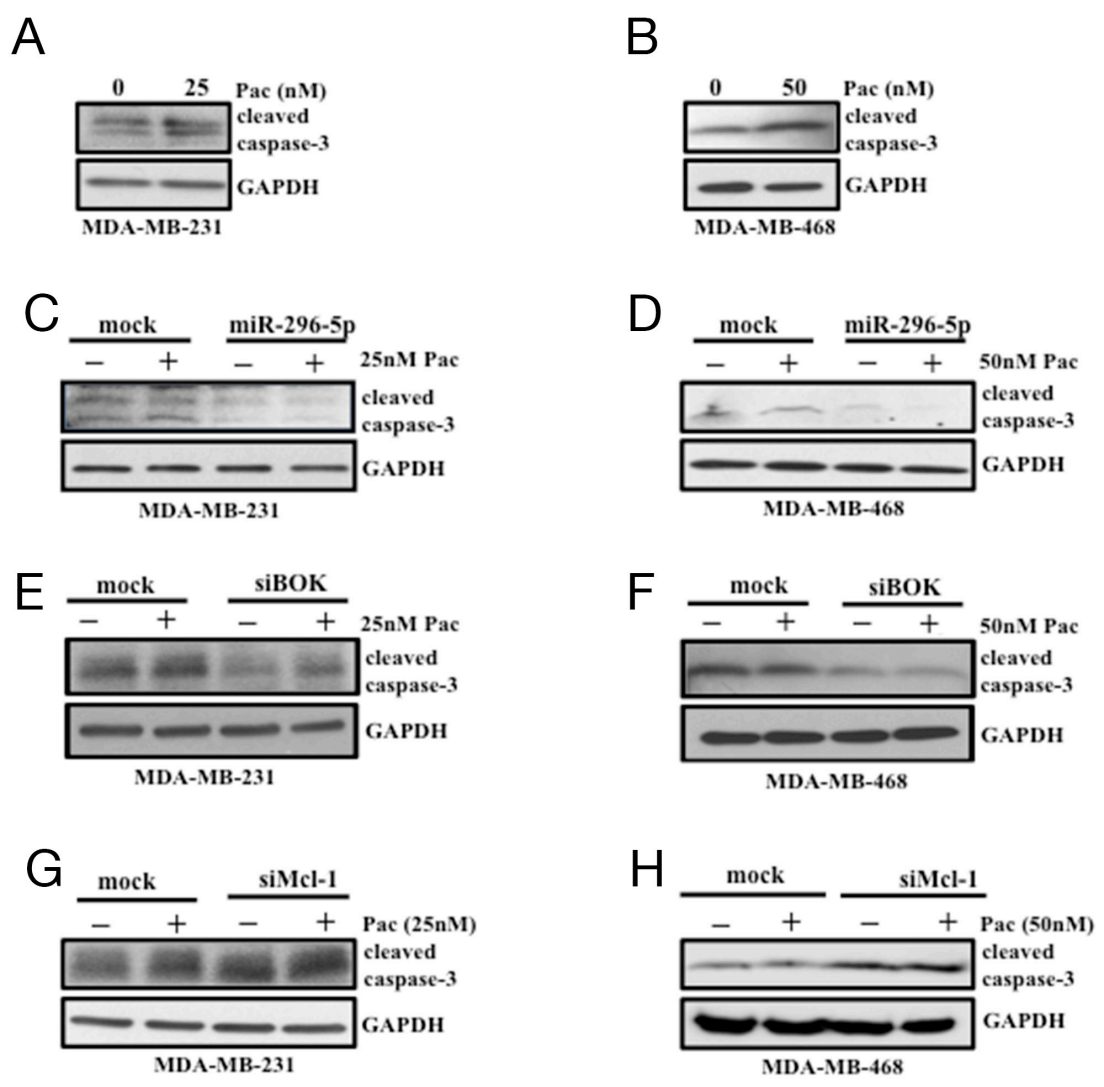
miR-296-5p and BOK silencing protect breast cancer cells from paclitaxel-induced apoptosis **(A, B)** Western blot analysis on MDA-MB-231 (A) and MDA-MB-468 cells (B) treated with vehicle or indicated dose of paclitaxel (Pac) using antibody against caspase-3. **(C, D)** Western blot analysis on MDA-MB-231 and MDA-MB-468 cells transfected with miR-296-5p in the presence and absence of paclitaxel (pac) using antibody against caspase-3. **(E, F)** Western blot analysis on MDA-MB-231 and MDA-MB-468 cells transfected with mock or BOK-siRNA in the presence and absence of paclitaxel (pac) using antibody against caspase-3. **(G, H)** Western blot analysis on MDA-MB-231 and MDA-MB-468 cells transfected with mock or Mcl-1-siRNA in the presence and absence of paclitaxel (pac) using antibody against caspase-3. Gel pictures are representative of three independent experiments.

Next, we assessed whether the compensatory effects of BOK and Mcl-1 silencing on breast cancer growth has similar effect on apoptosis. To address this, miR-296-5p or BOK or Mcl-1 knockdown cells were treated with paclitaxel, a chemotherapy drug that acts as a microtubule destabilizer and induces apoptosis [28] via caspase-3 activation [29]. Breast cancer cells treated with paclitaxel induced caspase-3 activation compared to control (Figures 6A, 6B). Notably, breast cancer cells transfected with either miR-296-5p or siRNA against BOK led to significant suppression of activated caspase-3 level in the presence of paclitaxel compared to vehicle treatment (Figures 6C–6F). In contrast, cleaved caspase-3 level was significantly elevated in Mcl-1 specific knockdown cells in the presence of paclitaxel when compared to vehicle treated cells (Figures 6G–6H). To further substantiate these results, we performed annexin V-FITC/PI staining on miR-296-5p or BOK siRNA transfected breast cancer cells. Consistent with our earlier results, miR-296-5p or BOK silencing did not induce apoptosis. Furthermore, in the presence of paclitaxel treatment, miR-296-5p and BOK silencing suppressed paclitaxel-induced apoptosis compared to scramble transfected breast cancer cells treated with paclitaxel (Supplementary Figure 6). These results suggested that miR-296-5p could protect cancer cells from paclitaxel-induced apoptosis via BOK.

### GSK3α/β regulates BOK expression in breast cancer cells

**Figure 7:**
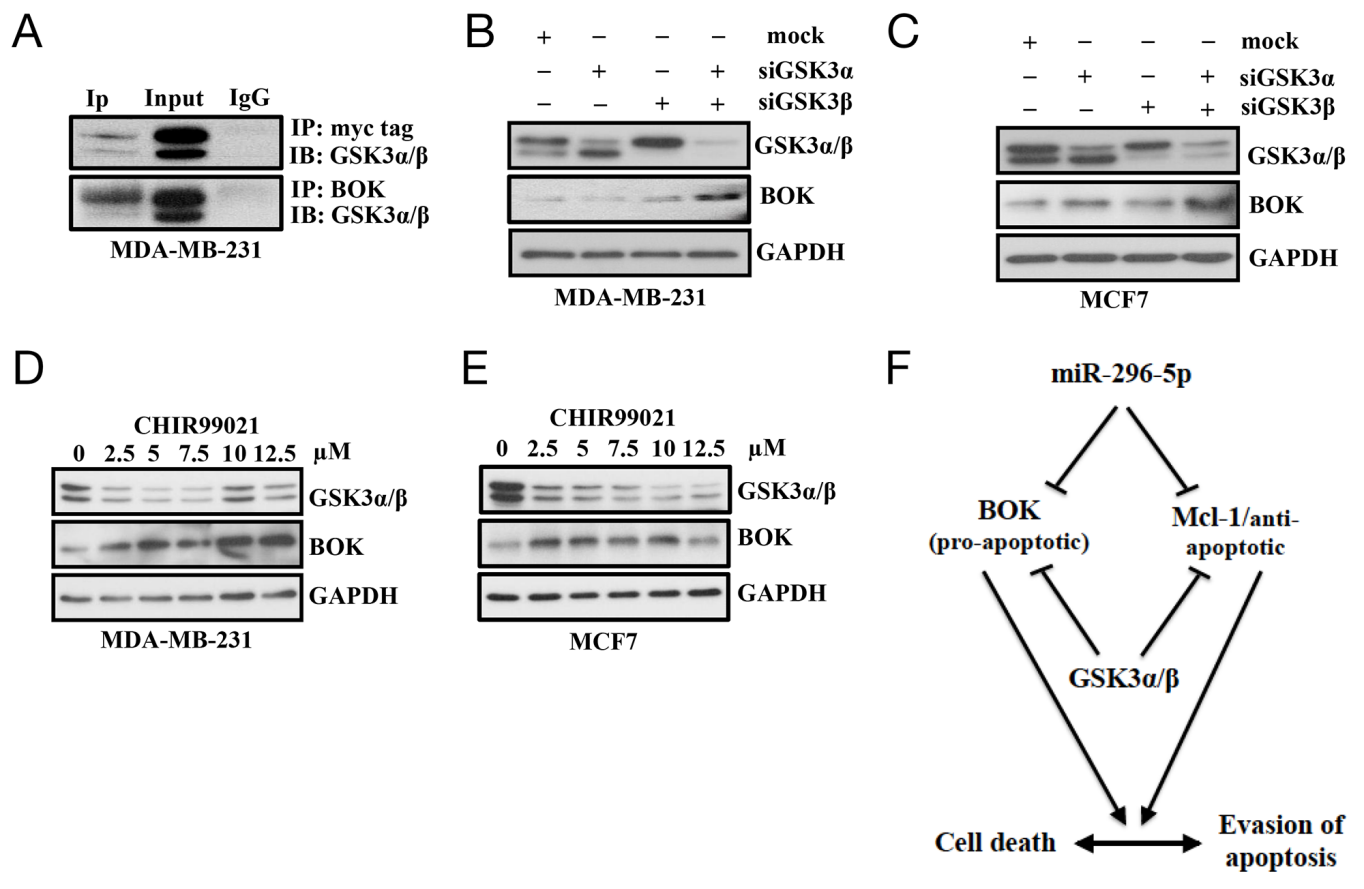
GSK3α/β regulates BOK expression **(A)** Immunoprecipitation on MDA-MB-231 cells transfected with control- or myc-tagged BOK expression vector using antibody against myc or BOK; and probed with (IB) with antibody against GSK3. Immunoprecipitation with IgG served as a negative control. **(B, C)** Western blot analysis on MDA-MB-231 (B) and MCF-7 (C) cells transfected with either mock, or GSK3α-siRNA, or GSKβ-siRNA, or GSK3α-siRNA + GSK3β-siRNA using antibodies against indicated proteins. GAPDH served as a loading control. **(D, E)** Western blot analysis on MDA-MB-231 (D) and MCF-7 (E) cells treated with vehicle control or increasing dose of GSK3 inhibitor CHIR99021 using antibody against indicated proteins. GAPDH served as a loading control. **(F)** Model showing regulation of pro- and anti-apoptotic proteins. Our results indicate that post-transcriptional regulation by miR-296-5p and post-translational regulation by GSK3 of pro-apoptotic and anti-apoptotic proteins is critical for determining the fate of cancer cells to survive or undergo apoptosis. Furthermore, our results indicate that expression of pro-apoptotic (BOK) and anti-apoptotic (Mcl-1) proteins is tightly regulated and relative ratio of these proteins is crucial to maintain the normal cellular homeostasis.

Having shown that BOK is a critical regulator that plays an important role in determining whether cancer cells die or survive, we wondered whether there are other means by which BOK expression may be regulated in breast cancers. One possibility is the “post-translational modification” as recent report demonstrated that BOK protein levels could be regulated via ubiquitin degradation pathway [24]. To begin to study that, we generated breast cancer cell lines stably expressing BOK. Interestingly, we observed elevated BOK mRNA but not BOK protein in our stable breast cancer cells. This result further supported the notion that BOK expression is regulated at the post-translational level. As protein phosphorylation is understood to regulate multitude of protein expression, we investigated whether BOK protein is phosphorylated by protein kinases. Using an *In silico* approach (http://www.cbs.dtu.dk/services/NetPhos/), we identified multiple sites where BOK can be potentially phosphorylated by kinases including protein kinase A (PKA), protein kinase C (PKC), and glycogen synthase kinase 3 (GSK3) (Supplementary Figure 7). For further analysis, we focused on GSK3 as it has been shown to be associated with mitochondrial apoptotic signal [30]. Moreover, GSK3 is known to phosphorylate other Bcl-2 members such as BAX [31]. The GSK3 gene family consists of GSK3α and GSK3β, each of which has distinct roles but are also known to compensate each other’s function [32]. Immunoprecipitation using antibody against myc-tag or BOK identified GSK3α/β as bonafide BOK interacting proteins (Figure 7A). Next, we directly tested whether GSK3α/β regulates BOK expression. Pharmacological inhibition of GSK3 with CHIR99021 or silencing of GSK3α/β using siRNAs resulted in elevated BOK protein levels in breast cancer cells (Figures 7B–7E). However, BOK mRNA levels did not show any significant change (data not shown) further confirming that GSK3 regulates BOK expression at the post-translational level. Future experiments using deletion constructs will identify potential sites in BOK protein that are phosphorylated by GSK or other kinases. Nevertheless, to our knowledge this is the first report to show that BOK expression is regulated at the post-translational level by GSK3.

### miR-296-5p and its target gene expression in breast cancers

We determined whether miR-296-5p expression correlated with BOK and Mcl-1 expression levels in breast cancer patients. Meta-analysis of TCGA data set revealed that like BOK, the levels of both miR-296 and Mcl-1 were significantly lower in breast cancer tissues compared to normal adjacent controls (Supplementary Figures 8A–8B). It is worth noting that available TCGA data set does not distinguish between miR-296-5p and miR-296-3p. The lower level of Mcl-1 in breast cancer specimens was unexpected given that Mcl-1 expression was previously shown to correlate with the tumor grade in breast cancer patients [33]. Since Ding. et al., compared Mcl-1 expression between tumors (not between control and tumors), it is possible that even though Mcl-1 expression may be associated with the aggressiveness of a sub-set of tumors, the Mcl-1 expression overall is lower in breast cancers compared to normal control. It is also possible that Mcl-1 expression pattern at the RNA and proteins levels are different in breast cancers as Ding et al., used immunohistochemical analysis to score Mcl-1 expression. Next, we addressed the clinical significance of miR-296-5p and Mcl-1 in breast cancer patients. Kaplan-Meir analysis using METABRIC and TCGA data sets showed that lower miR-296-5p (or miR-296 for TCGA) expression correlated with higher overall survival of breast cancer patients (Supplementary Figures 9A and 9B). This was an unexpected finding given that miR-296-5p acts as a tumor suppressor in breast cancers. It is possible that differential regulation of BOK and Mcl-1 by miR-296-5p may determine whether pro- or anti-apoptotic functions prevail and accordingly affect the survival of breast cancer patients. In accordance with that, higher Mcl-1 expression was correlated with lower overall survival and relapse-free survival of breast cancer patients as revealed by the Kaplan-Meir analysis (Supplementary Figures 10A–10B).

## DISCUSSION

The balance between pro-proliferation and pro-cell death signals determines the fate of cancer cells to survive/grow or die. The intrinsic apoptotic pathway and in particular Bcl-2 family members play a pivotal role in regulating this balance. For example, increased expression of anti-apoptotic Bcl-2 proteins that block the action of pro-apoptotic effectors (BAX and BAK) has been associated with cancer cell progression and resistance to pro-apoptotic signals [34]. Similarly, enhancing the signal of pro-apoptotic Bcl-2 homology domain 3 (BH3) proteins was the basis for the FDA approval of BH3 mimetics for treating chronic lymphocytic leukemia (CLL). In addition, albeit paradoxically, pro-apoptotic signal has been proposed to promote tumorigenesis [35]. These observations underline the importance of understanding the mechanisms by which expression of anti- and pro-apoptotic proteins may be regulated so that optimal therapeutic strategies for killing cancer cells can be developed.

In the present study, we focused on the regulation of BOK, one of the least studied and poorly understood pro-apoptotic Bcl-2 member. We demonstrated for the first time that BOK is regulated by miR-296-5p in breast cancers. Interestingly, we show that anti-apoptotic Mcl-1 is also regulated by miR-296-5p. Given that miR-296-5p is expressed at lower levels and acts as a tumor suppressor in breast cancers, our results suggest that the relative levels of BOK and Mcl-1 as well as other pro- and anti-apoptotic proteins may be critical for evading apoptosis and continued proliferation of breast cancer cells. This will be likely because miR-296-5p may have differential regulatory effect on BOK and Mcl-1 with BOK (in comparison to Mcl-1) being more sensitive to changes in levels of miR-296-5p. Indeed, our results reveal that there are at least sixteen putative miR-296-5p binding sites in BOK 3’-UTR compared to two in Mcl-1 3’-UTR. It is also possible that the absence of miR-296-5p could results in increased (or no change in) levels of other anti-apoptotic Bcl-2 proteins that bind to and neutralize the functions of pro-apoptotic proteins in breast cancer. Supporting this, we show that Bcl-2 and Bcl-xL are putative targets of miR-296-5p; and anti-apoptotic Bcl-2 proteins are known to inhibit mitochondrial outer membrane permeabilization (MOMP) by directly binding and activating pro-apoptotic BAX and BAK [36, 37]. These findings suggest that miR-296-5p may act as a key regulator that fine-tunes both pro- and anti-apoptotic signals. Consistent with that, our results showed that miR-296-5p, though acts as a tumor suppressor, protect breast cancer cells from paclitaxel-induced apoptosis. It is possible that under acute pro-apoptotic pressure (such as paclitaxel) miR-296-5p may preferentially attenuate BOK expression leading to resistance to drug-induced cell death and consequently cell survival. Indeed, previous studies have shown that loss of BOK promotes resistance to ER-stress-induced apoptosis *in vivo* [38]. Furthermore, similar to miR-296-5p, tumor suppressor miR-34c has also been reported to protect cancer cells to chemotherapy drug-induced apoptosis [39].

In addition to miR-296-5p, our study shows that BOK expression may be regulated via functional interaction with Mcl-1. Although BOK was initially identified as a Mcl-1 interacting protein, there was no evidence demonstrating regulatory or functional interaction between these two proteins. This study is the first to show that BOK and Mcl-1 regulate each other’s expression and function. Our results showing rescue of growth inhibitory effect of Mcl-1 knockdown in BOK and Mcl-1 depleted breast cancer cells suggest that BOK and Mcl-1 may be in a complimentary feedback loop. It is possible that simultaneous loss of BOK and Mcl-1 may prompt other pro-survival genes to compensate for their loss. Indeed, levels of several pro-survival proteins were found to be elevated when breast cancer cells were depleted for both Mcl-1 and BOK compared to silencing of either of them alone. Furthermore, since Mcl-1 is highly expressed in several cancers, our results support the notion that the increased levels of Mcl-1 may block BOK pro-apoptotic activity by interacting and sequestering BOK away from localizing to the mitochondria.

Our data indicate that post-translational modification may be another mechanism by which BOK expression is regulated. Our study is the first to show that BOK is a bonafide target of GSK3α/β. GSK3β has been shown to regulate the levels and function of its targets by phosphorylating and consequently setting them for either degradation via ubiquitination and proteolysis [40, 41] or by stabilizing their activities [42]. Therefore, it is likely that GSK3α/β-dependent phosphorylation of BOK may be one of the important mechanisms that control BOK expression and function in cancer cells. Supporting this, we identified several potential GSK3 phosphorylation sites on BOK protein and showed that the inhibition of GSK3α/β led to increased expression of BOK in breast cancer cells. Furthermore, a recent report demonstrated that BOK could be regulated by ubiquitin/proteasome-dependent pathway [24]. Given that GSK3 can act as a tumor promoter or suppressor and is reported to target other Bcl-2 family members including Bcl-2, Mcl-1 and BAX, it is plausible that stabilization of anti-apoptotic proteins (such as BCL2L12A) and degradation of pro-apoptotic proteins (such as BOK) by GSK-3 may induce tumor growth, while GSK3-mediated stabilization of pro-apoptotic protein (such as BAX) and degradation of anti-apoptotic proteins (such as Mcl-1) may lead to tumor suppression.

In summary, our study unveils novel mechanisms by which the levels and activities of pro-apoptotic protein BOK and anti-apoptotic protein Mcl-1 are regulated. Furthermore, our study attests that the regulation of pro- and anti-apoptotic Bcl-2 family member proteins is intertwined and a fine balance of their levels or activities or both is critical for determining whether cancer cell proliferate or undergo programmed cell death.

## MATERIALS AND METHODS

### Expression analysis in breast cancer specimens and survival analysis

Meta-analysis for BOK expression was performed on a public domain gene expression dataset from The Cancer Genome Atlas Research Network: http://cancergenome.nih.gov/. Kaplan-Meier survival analyses for the disease outcomes were performed using the online database (www.kmplot.com) and the percentiles of the patients using the upper were auto-selected based on the computed best performing thresholds as cutoffs. The *p-values* distributions of each comparison of cancer vs normal adjacent tissue obtained from differential gene expression analysis (see below) were considered to check for possible size effects.

### Cell lines, culture and reagents

Human breast cancer cell lines MDA-MB-231, MDA-MB-468, MCF7 were obtained from the American Type Culture collection (ATCC Manassas, VA). MDA-MB-231 and MCF7 cells were grown in 1X high glucose Dulbecco’s modified Eagle’s medium (DMEM) with sodium pyruvate (Invitrogen) supplemented 10% Fetal Bovine Serum (FBS), 1% nonessential amino acids, 1% Penicillin and streptomycine (P/S). MDA-MB-468 cells were grown in Roswell Park Memorial Institute medium (RPMI) 1640 which was supplemented with 10% FBS and 1% P/S. The cells were cultured in 5% CO_2_ at 37°C humidified incubator.

### RNA extraction and quantitation real-time PCR

Total RNA was isolated with Qiagen RNA extraction kit according to the manufacturer’s protocol. One microgram of total RNA was used for reverse transcription reaction using iScript Reverse Transcription kit (Bio-rad Hercules, CA) according to manufacturer’s protocol. The expression levels of BOK, or Mcl-1, and 18S (housekeeping gene) were analyzed using SYBR Green Master mix (Qiagen Valencia, CA) and gene-specific primers on Applied Biosystems (ABI) Thermocycler 7900 Fast. The real-time PCR was done in triplicate for each run. Reverse transcription was performed using the iScript Reverse Transcription kit (Bio-rad Hercules, CA). Real-time PCR was conducted on an ABI Prism 7900 Fast Sequence Detection System (ThermoFisher Scientific Inc., Foster City, CA) using 95°C denaturation for 15 minutes followed by 40 cycles of 94°C for 15 seconds, then 60°C for 30 seconds, and 72 °C for 30 second. Fold change was generated using the equation 2^−ΔΔCt^.

### Cell transfection

Prior to transfection, cells were seeded in 10% FBS medium with antibiotics and incubated overnight at 37°C in 5% CO_2_. Subsequently, the medium was replaced with the medium containing 10% FBS without antibiotics. Transfection complex was prepared with RNAiMax for siRNA transfection or Lipofectamine 2000 for plasmid transfection in 1X Opti-MEM using manufacturer’s recommendation. Breast cancer cells were transfected with 75 nM of pre-miR-296-5p (Ambion, USA) or 100 nM of anti-miR-296-5p or mock (containing transfection reagent in 1X Optimem medium) or un-transfected control. For gene-specific knockdown, breast cancer cells were transfected with 75 nM of siRNA (Sigma-Aldrich) or scramble-siRNA or mock.

### Western blot

Cells were rinsed with 1X PBS and lysed in RIPA buffer and the lysate was incubated on ice for 30 minutes and centrifuged at 4°C for 10 minutes at maximum. After the centrifugation, cell supernatant was transferred into a new 1.5 mL microfuge tube. The protein concentration was quantified using 1X Bradford assay (Bradford, Hercules CA). Fifty microgram of proteins were denatured in sample buffer and separated by SDS-PAGE and transferred to PVDF-membranes (Millipore, MS). Membranes were blocked with 5% milk, washed, incubated in appropriate primary antibodies. Membranes were then incubated with horseradish peroxidase-conjugated secondary antibodies for 1 hour at room temperature, washed 3X for 15 minutes and developed using ECL chemiluminescent kit (Millipore, Billerica MS). Polyclonal rabbit anti-BOK (cat.# SAB1300048) was purchased from Sigma Aldrich, St. Louis, MO. Mouse anti-GAPDH (cat.# sc-32233) was purchased from Santa Cruz Biotechnology. Pro-apoptosis Bcl-2 family antibody (cat.# 9942), anti-Bcl-2 (cat.# 15071), Anti-Mcl-1 (cat.# 4572) and anti-Bcl-xL (cat.# 2764) were purchased from Cell signaling Technology. Antibody against caspase-3 (cat.# sc-7148) was purchased from Santa Cruz Biotechnology.

### Colony formation assay

Breast cancer cells were transfected with miR-296-5p, anti-miR-296-5p, mock and subjected to colony formation assay as described previously [43]. Briefly, 800 cells of MDA-MB-231 or 2,000 cells of MDA-MB-468, and MCF7 were plated in 2 mL high glucose 1X DMEM supplemented with 10% FBS, 1% P/S and incubated at 37°C and 5% CO_2_ for 8 days and colonies were then stained in crystal violet dye (0.5% crystal violet and 20% methanol).

### Migration and invasion

Breast cancer cells transfected with mock, miR-296-5p or anti-miR-296-5p were subjected to migration and invasion assay as described previously [43].

### Plasmids

BOK cDNA plasmid was obtained from DNASU Plasmid Repository (Phoenix, AZ). The cDNA was excised from pDNR-Dual vector using SalI and HindIII restriction enzymes and subcloned into pGEM vector to maintain the orientation. PGEM-BOK construct was digested with BamHI and HindIII, and cloned into pCMV6 mammalian expressing vector.

### Luciferase assays

MDA-MB-231 cells were transfected using lipofectamin 2000 (ThermoFisher Scientific, Grand Island, NY) according to the manufacturer’s specification. A total of 50,000 cells were seeded in each well in six-well plate and incubated overnight. The cells were then co-transfected with renilla luciferase vector (pRL-null) and firefly luciferase vector containing pGL3-wt-BOK, or pGL3-mut-1-BOK, or pGL3-mut-2-BOK, or pGL3, or pGL3-wt-Mcl-1, or pGL3-mut-Mcl-1 overnight and incubated in fresh complete medium for an additional 48 hours after transfection. The transfected cells were then transfected with miR-296-5p or mock and incubated for additional 24 hours. Next, cells were harvested with 1X passive lysis buffer (Promega Madison, WI) and the luciferase activities were read using GLOMAX 20/20 luminometer (Promega Madison, WI).

### Apoptosis analysis

MDA-MB-231 cells were seeded in 12-well tissue culture plates (5 × 10^5^ cells/well). On the second day, the cells were transfected with either miR-296-5p or scramble or siRNA against BOK or Mcl-1 and incubated for overnight. The medium was changed and cells were incubated for additional 24 hours. Later, the cells were either treated with 12.5nM paclitaxel or with vehicle control for 72 hours. After treatment, the cells floating in the medium were collected. The adherent cells were detached with 0.05% trypsin. Then the culture medium containing FBS and floating cells was added to inactivate the trypsin. After being pipetted gently, the cells were centrifuged for 5 min at 1500× *g*. The supernatant was removed and the cells were stained with annexin V-FITC and PI according to the manufacturer’s instructions. Untreated cells were used as control for the double staining. The cells were analyzed immediately after staining using a FACScan flow cytometer and FlowJo 9.0 software. For each measurement, at least 20,000 cells were counted.

### Statistical analysis

Results are presented as means of ± SD. Statistical comparisons between two groups of data were made using two-way ANOVA. The *p*-Value of < 0.05 is denoted as *, *p*-Value of < 0.01 as **, *p*-Value of < 0.001 denoted as ***.

## SUPPLEMENTARY MATERIALS FIGURES


