# Supplementary material for: Novel post-transcriptional and post-translational regulation of pro-apoptotic protein BOK and anti-apoptotic protein Mcl-1 determine the fate of breast cancer cells to survive or die
Source: Oncotarget. 2017 Sep 12;8(49):85984–96. doi: 10.18632/oncotarget.20841 (PMC5689661; doi:10.18632/oncotarget.20841)
Supplement: Supplementary file 1 [file oncotarget-08-85984-s001.pdf]

Novel post-transcriptional and post-translational regulation of pro-apoptotic protein BOK and anti-apoptotic protein Mcl-1 determine the fate of breast cancer cells to survive or die

SUPPLEMENTARY MATERIALS

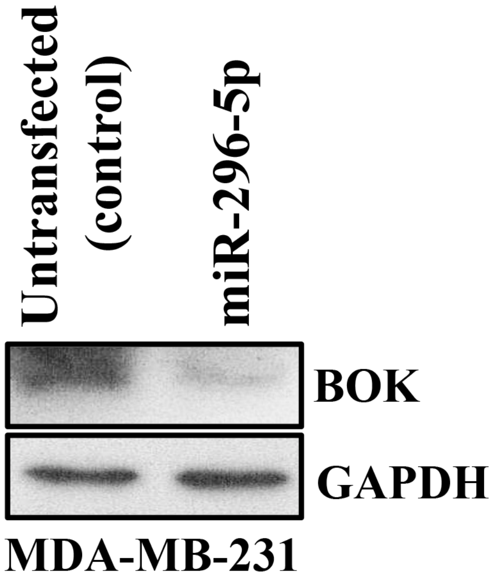

Supplementary Figure 1: miR-296-5p suppresses BOK expression. Western blots analysis of BOK protein level in untransfected control and miR-296-5p-transfected MDA-MB-231 cells using antibody against BOK. GAPDH served as loading control. Gel photograph is representative of three independent experiments.

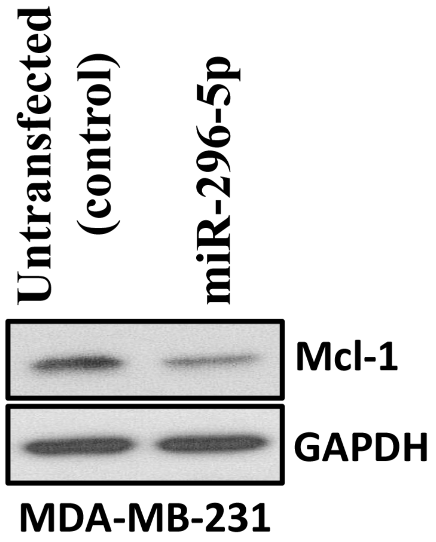

Supplementary Figure 2: miR-296-5p overexpression attenuates Mcl-1 expression. Western blot analysis of Mcl-1 protein level in untransfected control and miR-296-5p-transfected MDA-MB-231 cells using antibody against Mcl-1. GAPDH was used as loading control. Gel photograph is representative of three independent experiments.

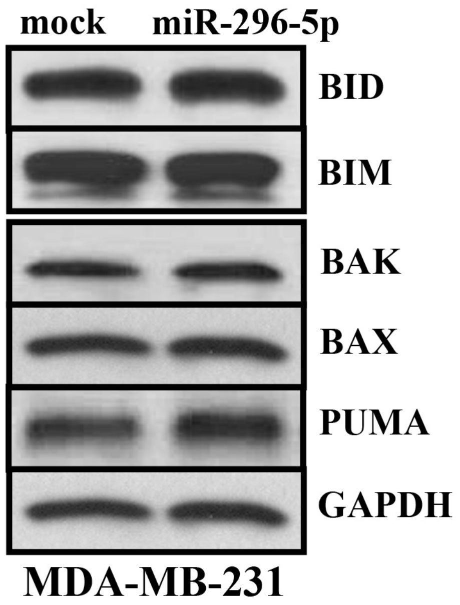

Supplementary Figure 3: miR-296-5p does not affect expression of several pro-apoptotic Bcl-2 member proteins. Western blots analysis of MDA-MB-231 cells transfected with mock or miR-296-5p using antibodies against indicated proteins. GAPDH was used as a loading control. Gel photograph is representative of three independent experiments.

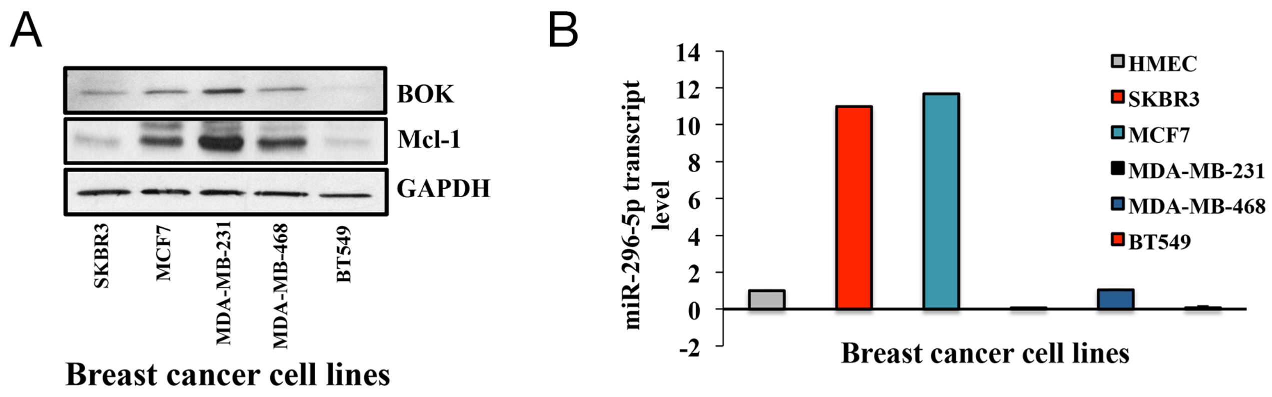

Supplementary Figure 4: miR-296-5p and BOK expression in breast cancer cell lines. **(A)** Western blots analysis of BOK protein levels in different breast cancer cell lines using antibody against BOK. GAPDH served as the loading control. **(B)** Real time PCR analyses of miR-296-5p expression in breast cancer cell lines. Result was normalized with miR-296-5p expression in HMEC cells. U19 RNA was used as the loading control.

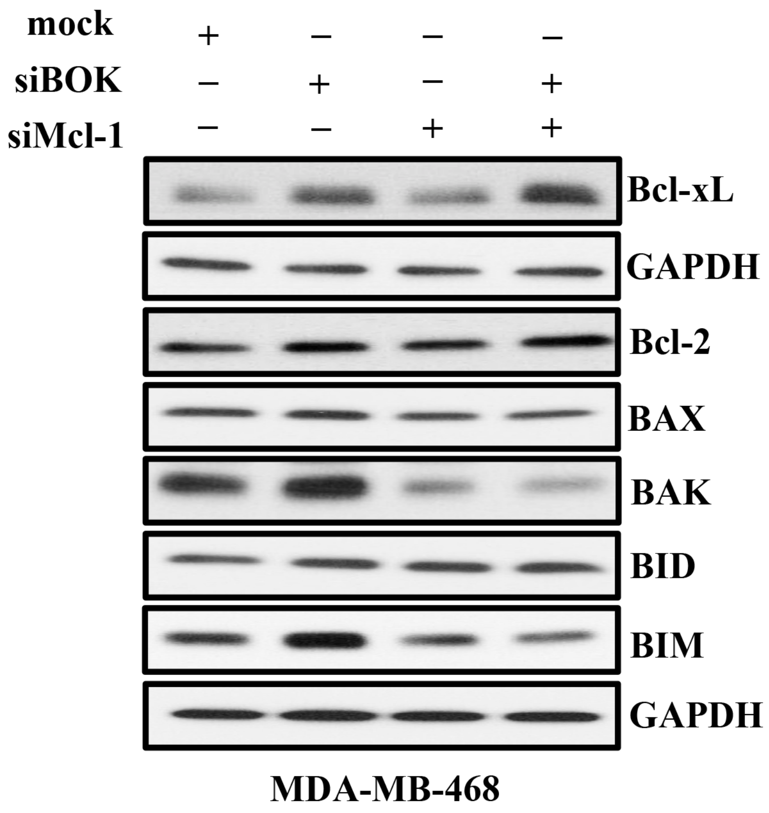

Supplementary Figure 5: Pro- and anti-apoptotic proteins constitute compensatory feed- back loop. Western blot analysis of anti-apoptotic and pro-apoptotic Bcl-2 proteins in mock, BOK-siRNA, Mcl-1-siRNA-, or Mcl-1 and BOK-siRNAs transfected MDA-MB-468 cells using antibodies against indicated proteins. GAPDH was used as a loading control. Gel photograph is representative of three independent experiments.

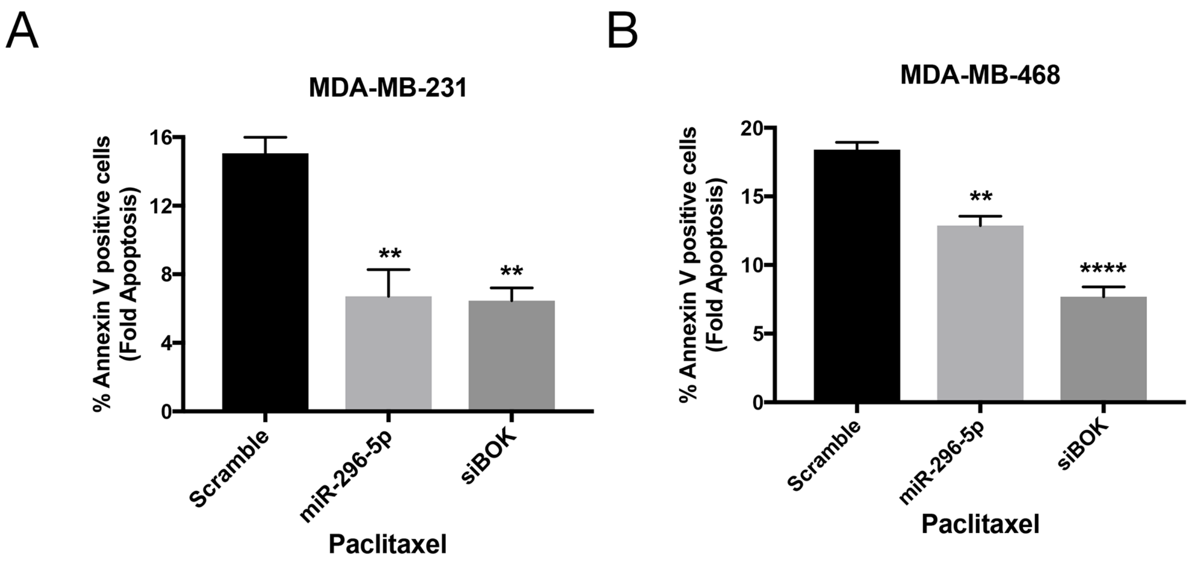

Supplementary Figure 6: BOK silencing suppresses paclitaxel-induced apoptosis in breast cancer cells. (A, B) Annexin-FITC analysis of apoptotic cells in scramble, miR-296-5p or BOK-siRNA-transfected MDA-MB-231 (A) and MDA-MB-468 (B) cells in the presence or absence of paclitaxel. MDA-MB-231 and MDA-MB-468 were treated with either 12.5nM and 50nM paclitaxel, respectively, or vehicle for 72 hours. Apoptosis was quantified using flow cytometry analysis after staining with annexin V/PI. Results were normalized with their respective untreated control and plotted as fold change of Annexin V positive cells. Data are presented as the mean ± S.E. of triplicate experiments. **** $p < 0.0001$ *vs.* control.

BOK protein sequence

```
     MEVLRRSSVFAAEIMDAFDRSPTDKELVAQAKALGREYVHARLLRAGLSW    #    50
     SAPERAAPVPGRLAEVCAVLLRLGDELEMIRPSVYRNVARQLHISLQSEP    #   100
     VVTDAFLAVAGHIFSAGITWGKVVSLYAVAAGLAVDCVRQAQPAMVHALV    #   150
     DCLGEFVRKTLATWLRRRGGWTDVLKCVVSTDPGLRSHWLVAALCSFGRF    #   200
     LKAAFFVLLPER                                          #   250
%1   ......SS............S.T.........................S.    #    50
%1   S...............................S.Y............S..    #   100
%1   ..............S...T.....S.........................    #   150
%1   .........T..T................................S....    #   200
%1   ............
```

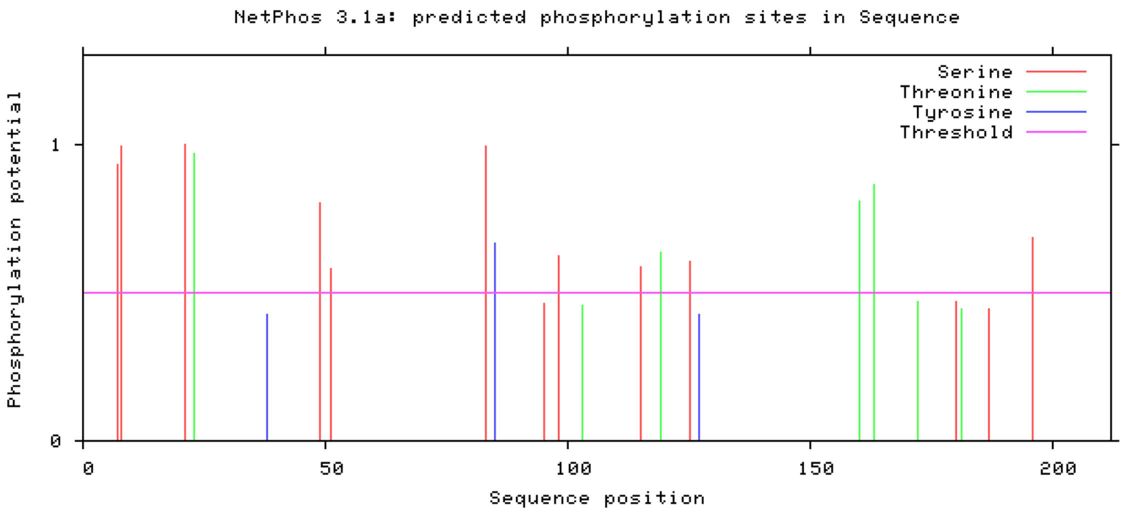

Supplementary Figure 7: Putative phosphorylation sites in BOK protein. Schematic representation of BOK protein sequence showing putative phosphorylation sites of different protein kinases as predicted by NetPhos 3.1a [2].

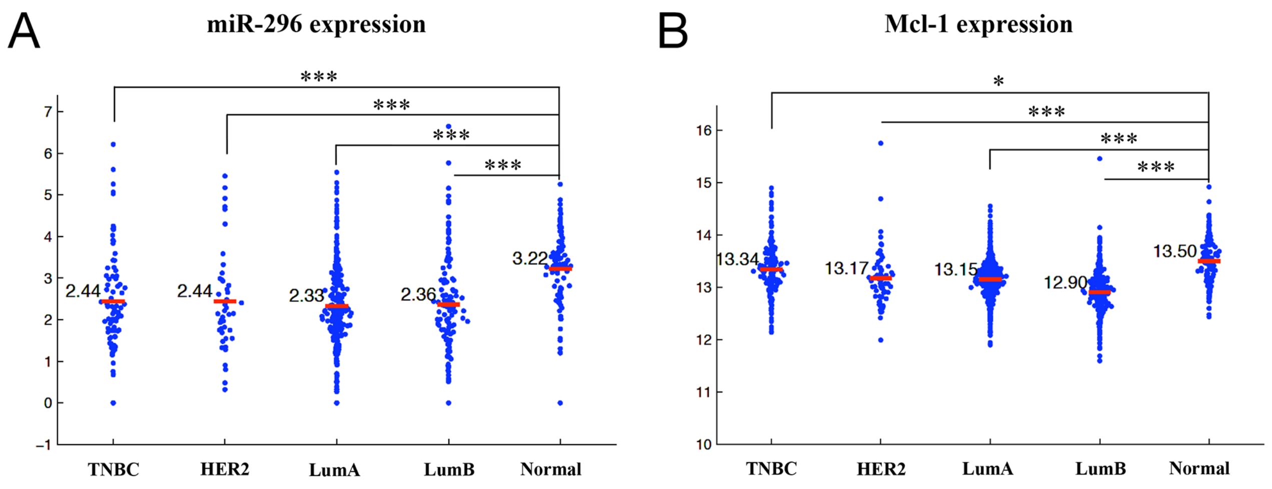

Supplementary Figure 8: Decreased miR-296 and Mcl-1 expression in breast cancers. (A, B) Meta-analysis of miR-296 and Mcl-1 expressions in TCGA data set for normal adjacent control and human breast cancer tissue specimens. Patients were stratified into 'low' and 'high' BOK expression based on upper quartile as cutoff. * *p < 0.05;* *** *p* < 0.001 *vs.* control.

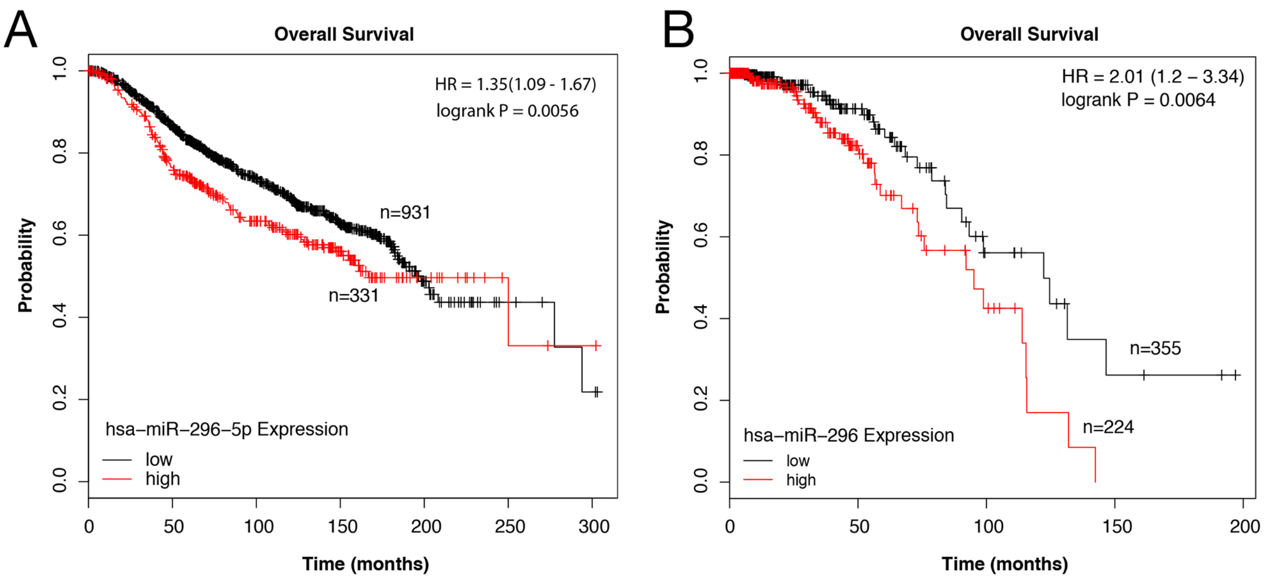

Supplementary Figure 9: Increased miR-296-5p expression correlates with poor disease outcome in breast cancer patients. (A, B) Kaplan-Meier analyses of overall survival of breast cancer patients in using KM plotter database (www.kmplot.com) [1]. *p-values* were calculated with logrank (Mantel-Cox) test. Patients were stratified into 'low' and 'high' miR-296-5p or miR-296 expression based on upper quartile as cutoff. The results shown here are based upon the data generated by the TCGA Research Network: http://cancergenome.nih.gov/.

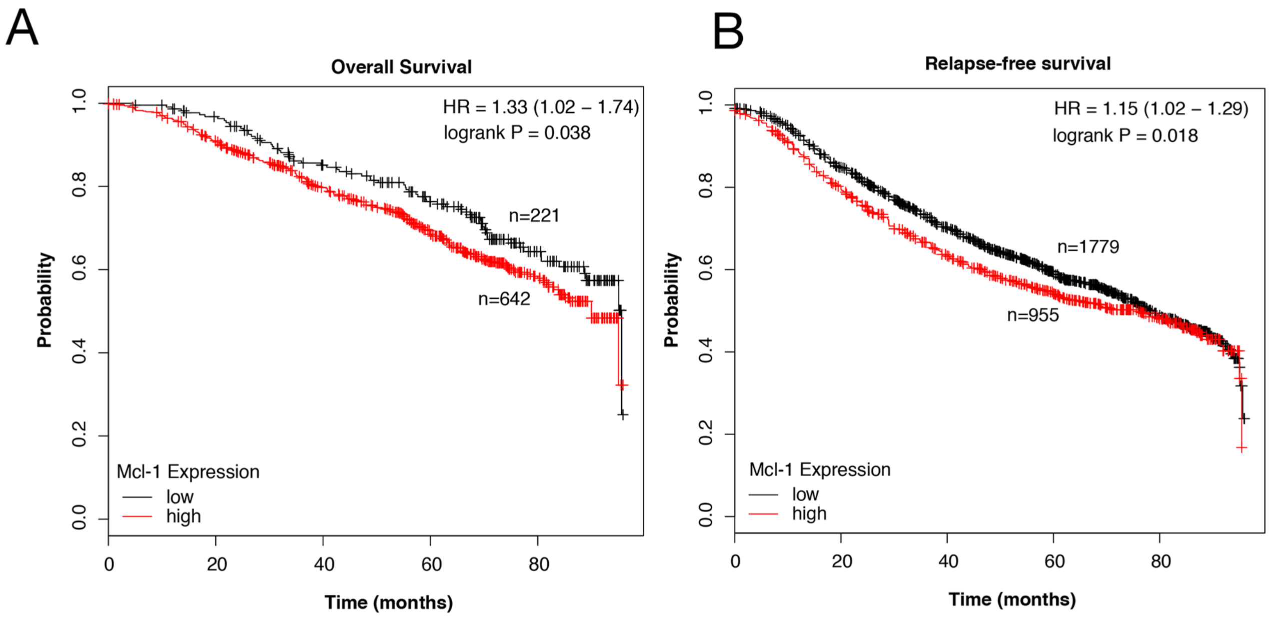

Supplementary Figure 10: Increased Mcl-1 expression correlates with increased mortality in breast cancers patients. **(A, B)** The Kaplan-Meier analyses of overall survival and relapse free-survival of breast cancer patients using KM plotter database (www.kmplot.com) [1]. *p-values* were calculated with logrank (Mantel-Cox) test. Patients were stratified into 'low' and 'high' Mcl-1 expression based on upper quartile as cutoff. The results shown here are based upon the data generated by the TCGA Research Network: http://cancergenome.nih.gov/.

REFERENCES

1. Győrffy B, Lanczky A, Eklund AC, Denkert C, Budczies J, Li Q, Szallasi Z. An online survival analysis tool to rapidly assess the effect of 22,277 genes on breast cancer prognosis using microarray data of 1,809 patients. Breast Cancer Res Treat. 2010; 123:725–31.

2. Blom N, Gammeltoft S, Brunak S. Sequence and structure-based prediction of eukaryotic protein phosphorylation sites. J Mol Biol. 1999; 294:1351–62.
